# Medical Opinion and Movement

**Published:** 1907-06-08

**Authors:** 


### Medical Opinion and Movement.

Heroin has acquired in recent years a considerable reputation as a respiratory sedative. Chemically it is diacetyl-morphine, two of the hydrogen atoms of morphine being replaced by acetic radicals ($C_2H_3O$). According to Saint Martin and others who have studied its physiological action, this substitution product of morphine is actually more toxic and more dangerous in its effects than morphine itself. It has a weaker action upon the cerebral centres, but its convulsive and paralysing effect on the bulbo-medullary centres is greater. It is fifteen times more toxic than morphine for the rabbit, four and a half times more toxic for the dog, and thirty times more toxic for the donkey. Duhem, in a recent paper on the subject, gives his experience of sixteen cases in which the heroin habit had been acquired. He is of opinion that its use leads more easily to the drug habit than morphia, and that once the habit has been acquired it is much more difficult to cure. During treatment the patient reacts much as a morphomaniac would, but feebleness and prostration are more intense, and respiratory syncope is more liable to supervene. These facts should certainly act as a warning against a too free use of the drug, or a false notion of its innocence.

The report has just been issued of the Committee appointed by the Home Secretary to inquire into and report upon those diseases and injuries, other than injuries by accidents, that are due to industrial employment, and should be added to the diseases enumerated in the third schedule of the new Workmen's Compensation Bill. The Committee encountered some difficulty in certain cases in drawing a distinct line between accidents and disease, and they took as the distinguishing feature the suddenness with which any injurious effects were produced. A further consideration which largely determined the recommendation of the committee was whether the disease or injury was so specific to the employment that its causation by the employment could be established in individual cases. The difficulty of establishing such a causation in cases of fibroid phthisis and bronchitis has led the committee not to recommend the immediate addition of this disease to the schedule. At a recent meeting of the Staffordshire pottery manufacturers it was resolved that the inclusion of "potters' asthma" in the schedule would act disastrously against employers and operatives alike, and that the disease should be dealt with by methods of prevention rather than compensation. This decision of the Committee therefore will doubtless give satisfaction to the trade. Many conditions of industrial poisoning have been recommended for compensation, and also such conditions as miners' nystagmus, glanders, compressed-air illness, miners' elbow and knee.

Some interesting papers have recently appeared in the "Journal of the Royal Army Medical Corps" on anti-typhoid inoculation in the Army, which afford convincing facts and arguments in proof of its protective effect against the disease, and of its importance as a prophylactic measure for the Army in India. The vaccine that has been used is that of Sir H. E. Wright, with some modifications. Soon after it was brought before the notice of the profession by Sir A. E. Wright in 1897, it was used on a large scale in Egypt and India with satisfactory results. Statistics obtained from the hospitals in South Africa during the Boer war showed a much smaller incidence of the disease on the inoculated than on the non-inoculated. The German Army physicians report favourably on its use in the Herrero campaign. In an epidemic which broke out among the 17th Lancers at Meerut in 1905 its efficacy was clearly demonstrated. Of 63 cases of typhoid 61 were in men who were not inoculated, and the remaining two were in men who had refused the second inoculation. It has been determined that the best results are obtained by two inoculations at an interval of ten days. The dosage has been fixed at .5 c.c. of vaccine containing 500,000,000 bacteria for the first inoculation, and double this dose for the second. This amount is estimated to give the maximum quantity of protective substances with the minimum severity of reaction. The reaction consists in local inflammatory signs at the seat of inoculation, together with general feverish symptoms lasting about 36 hours.

Dr. T. Stacey, of the Birmingham General Hospital, has made a series of studies on the significance of variations in the level of the diaphragm. In order to determine the level of the diaphragm, the author prefers to rely upon percussion of the gastric resonance, the upper limit of which normally lies in the sixth left intercostal space. Important indications are also obtained by percussion of the area of liver dulness, but Dr. Stacey does not consider the upper level of this dulness such a certain guide in regard to the level of the diaphragm as the signs just noted. The chief interest in his paper, however, lies in the significance which he attaches to an elevation of the diaphragm above the normal level. He lays down the proposition that "When, from any cause, the total volume of blood in circulation is materially diminished, the total bulk of the intrathoracic viscera is correspondingly diminished by the relative emptiness of the thoracic blood-vessels, especially those of the lungs. This diminution in the bulk of the intrathoracic contents shows itself by an elevation of the diaphragm, which has to be maintained at a higher average level than the normal in order to adjust the cubic contents of the thorax to the altered volume of its contained viscera." Dr. Stacey supports this proposition by several cases in which he observed a considerable elevation of the diaphragm above the normal level, and at the same time had good reason to suppose that there was a corresponding diminution in the volume of the blood. The cases included examples of asthenia following enteric fever, severe hæmorrhage, low diet, and anæmia. If these observations receive further confirmation, a "high diaphragm" should be a useful clinical sign in these conditions.

